# Potential application of item-response theory to interpretation of medical codes in electronic patient records

**DOI:** 10.1186/1471-2288-11-168

**Published:** 2011-12-16

**Authors:** Alex Dregan, Andy Grieve, Tjeerd van Staa, Martin C Gulliford

**Affiliations:** 1 Division of Primary Care and Public Health Sciences, King's College London, 42 Weston Street, London, SE1 3QD, UK; 2 Division of General Practice Research Database, Medicines and Healthcare Products, Regulatory Agency, 151 Buckingham Palace Road, London, SW1W 9SZ, UK; 3 Utrecht Institute for Pharmaceutical Sciences, Utrecht University, 3508 Utrecht, the Netherlands

## Abstract

**Background:**

Electronic patient records are generally coded using extensive sets of codes but the significance of the utilisation of individual codes may be unclear. Item response theory (IRT) models are used to characterise the psychometric properties of items included in tests and questionnaires. This study asked whether the properties of medical codes in electronic patient records may be characterised through the application of item response theory models.

**Methods:**

Data were provided by a cohort of 47,845 participants from 414 family practices in the UK General Practice Research Database (GPRD) with a first stroke between 1997 and 2006. Each eligible stroke code, out of a set of 202 OXMIS and Read codes, was coded as either recorded or not recorded for each participant. A two parameter IRT model was fitted using marginal maximum likelihood estimation. Estimated parameters from the model were considered to characterise each code with respect to the latent trait of stroke diagnosis. The location parameter is referred to as a calibration parameter, while the slope parameter is referred to as a discrimination parameter.

**Results:**

There were 79,874 stroke code occurrences available for analysis. Utilisation of codes varied between family practices with intraclass correlation coefficients of up to 0.25 for the most frequently used codes. IRT analyses were restricted to 110 Read codes. Calibration and discrimination parameters were estimated for 77 (70%) codes that were endorsed for 1,942 stroke patients. Parameters were not estimated for the remaining more frequently used codes. Discrimination parameter values ranged from 0.67 to 2.78, while calibration parameters values ranged from 4.47 to 11.58. The two parameter model gave a better fit to the data than either the one- or three-parameter models. However, high chi-square values for about a fifth of the stroke codes were suggestive of poor item fit.

**Conclusion:**

The application of item response theory models to coded electronic patient records might potentially contribute to identifying medical codes that offer poor discrimination or low calibration. This might indicate the need for improved coding sets or a requirement for improved clinical coding practice. However, in this study estimates were only obtained for a small proportion of participants and there was some evidence of poor model fit. There was also evidence of variation in the utilisation of codes between family practices raising the possibility that, in practice, properties of codes may vary for different coders.

## Background

Electronic patient records (EPRs) from primary care databases are increasingly used in health services and public health research but the analysis and interpretation of coded records has received little systematic study. It is common practice to identify cases of a condition of interest by determining whether one or more diagnostic codes, from a set of codes characterizing the condition, is ever recorded in that individuals' record. For acute conditions, each new occurrence may be identified as an episode of illness; for long-term conditions, the first occurrence of any code is usually used to identify cases of the condition.

There is often a need to confirm the validity of diagnostic classifications [1]. One strategy is to seek supporting information from within the EPRs. For example, diagnoses of stroke or myocardial infarction might be supported if hospital admissions and appropriate investigations were used around the time of diagnosis [2-4]. Another strategy is to review detailed paper-based records to seek clinical evidence that supports the diagnostic classification established within the EPRs [5,6]. This process is usually costly and logistically difficult and clinical records may only be reviewed for a sample of cases.

This paper explores a different potential approach to the interpretation of EPRs. This is based on the epidemiological analysis of occurrences of medical codes for the condition of interest. The suggested approach is grounded in psychometric theory. The classification of interest is regarded as a latent trait. The medical diagnostic codes that are selected to define the condition of interest are regarded as items. Each code may be affirmed if it occurs in the EPR, while it is not affirmed if there are no occurrences of the medical code in the EPR. Item Response Theory (IRT) models utilise item or code occurrences as outcomes and estimate parameters that characterise the properties of an item or code. This study explored the feasibility and utility of utilising IRT models to estimate code location parameters that characterize the probability of a medical code being endorsed by health professional as a function of patient's underlying medical condition [7].

## Methods

### The General Practice Research Database

The UK General Practice Research Database (GPRD) is an anonymised database containing EPRs from UK family practices. Data collected include demographics, medical diagnoses, prescription information, referral and treatment outcomes. Family practices included in the GPRD are broadly representative of all family practices in the United Kingdom in terms of geographical distribution, practice size and the age and gender distributions of registered patients. The quality of the information in the database is routinely checked for data accuracy and validity and has been found to be satisfactory for health research [8]. At the start of the database in 1987, family physicians contributing to the GPRD used a modified version of the Oxford Medical Information Systems coding system (OXMIS) to record diagnoses, but in recent years the Read coding system was used by all family practices. In order to make the study findings relevant to the current practice the present analyses were restricted to Read codes.

### Data source

This paper drew on our previous research and considered diagnostic coding for stroke [1]. The dataset comprised 48,239 individuals identified with a first stroke event between 1997 and 2006. All study patients had at least 24 months of up-to-standard follow-up prior to the date of the incident diagnosis of stroke. After excluding cases where date of death was before the first stroke index date, 47,845 individuals were identified for whom a first stroke index date was recorded between 1997 and 2006. All of these stroke participants were included in the descriptive analyses. Descriptive data of the sample have been reported previously [1,9]. The stroke participants were registered at 414 practices throughout the UK. There were 202 Read and OXMIS medical codes identified in our previous study [1]. There were 79,874 occurrences of the 202 codes among the 47,845 participants. Dummy variables were set up, one for each medical diagnostic code, to denote whether the code was recorded.

### Analysis

This study utilized a two-parameter logistic (2-PL) item response theory model [10]. In the 2-PL model, the probability of the electronic medical record of subject, *s*, containing an occurrence of a code, *i*, is estimated from the difference between the location parameter of that code, *βi*, and the trait level of subject, *θs*. The code location parameter is commonly referred to as the difficulty parameter. The two parameters characterise the relationship between the code and an underlying latent trait, in this case the degree of confidence in a stroke diagnosis. The code location parameter locates the position of the item in relation to the latent trait. When a code with a higher location parameter is endorsed this may indicate greater confidence in a stroke diagnosis. The 'discrimination' parameter (*αi*) denotes the capacity of the code to discriminate among subjects who are separated by only small differences in trait level. The impact of the difference between the subject's trait level and the item location on the probability of a code being affirmed, is lower for less discriminating items. Thus the probability that subject *s *has an instance of code *i *in his electronic record is given by:

$$\begin{array}{c} \text{P}\left(\text{Xis }=\text{ 1 }|\theta \text{s},\;\beta \text{i},\;\alpha \text{i}\right)\;=\\ =\text{ exp}\left(\alpha \text{i}\left(\theta \text{s }-\beta \text{i}\right)\right)∕\text{1}+\text{exp}\left(\alpha \text{i }\left(\theta \text{s }-\beta \text{i}\right)\right) \end{array}$$

The 2-PL model was implemented in the BILOG-MG program using marginal maximum likelihood estimation [11]. Parameters were estimated for 110 Read medical codes. The 2-PL model gave a substantially better goodness-of-fit than the one parameter logistic model (χ^2 ^= 352,873, _df = 109, p < 0.001). In addition, inspection of the correlation between each code and the overall construct suggested that the codes were not equally correlated which suggests that the 2-PL model provides a better fit the data [12]. As the last change across iteration was less than the convergence criterion (0.01) and the number of executed Newton (2) and EM cycles (20) was less than their maxima, the estimation process was judged to have reached convergence.

## Results

A total of 47,845 stroke participants were included in the analyses with 79,874 records of stroke codes, after excluding duplicate records on the same date. The distribution of respondents according to the number of stroke codes recorded during the study period is presented in Table 1.

**Table 1:** The distribution of patients according to the number of stroke codes registered over the study period (n = 47,845).

Number of stroke codes	N	Percentage
One	36,857	77
Two	8,277	17
Three	2,151	5
Four	446	1
Five	91	< 1
Six	21	< 1
Seven	2	< 1

Intraclass correlation coefficients (ICC) by family practice are shown in Table 2. There was considerable variation between practices in use of stroke codes especially for more frequently utilised codes including 'Stroke/CVA unspecified' code (ICC 0.25), 'Stroke annual review' (0.22) and 'Stroke monitoring' (0.16). There was limited between-practice variation for infrequently used stroke codes such as 'subarachnoid haemorrhage' (0.00).

**Table 2:** Intraclass correlation coefficients indicating practice-level variation in the recording of the commonest stroke codes (n = 79,847).

GPRD		Overall	Relative frequency^2^
207099	CVA unspecified	0.15	17%
234279	Stroke/CVA unspecified	0.25	24%
243296	Cerebrovascular accident unspecified	0.05	3%
213214	Stroke monitoring	0.16	14%
341089	Annual review	0.22	9%
234277	Cerebral infarction	0.04	3%
289093	Subarachnoid haemorrhage	0.00	3%
234275	Cerebral arterial occlusion	0.12	2%
298378	CVA- cerebral artery occlusion	0.08	3%
288824	Hemiparesis	0.01	3%
288823	Hemiplegia	0.01	1%
261654	Intracerebral haemorrhage	0.01	2%
225136	CVA due to intracerebral haemorrhage	0.05	2%
285273/257802	H/O Stroke or CVA	0.04	3%

### Item parameter estimates

For IRT analyses, the sample was restricted to those stroke participants for whom a Read medical code was used to register a stroke event (n = 45,619). Parameter estimates were obtained for 77 codes that were used in 1,942 participants accounting for about 4% of the total number of strokes. However, it should be noted that these parameter estimates were obtained through analysis of data for all subjects. The remaining, more frequently used codes, were automatically excluded from the estimation process because the correlation between individual and the sum of all codes was below the program's criterion (-0.15). These codes are judged to be out of the measurable range and not interpretable in the model [13]. For ease of presentation due to large number of stroke codes, the abridged parameter estimates from the 2-PL model for Read stroke codes recoded within 30 days of the index date are presented in Table 3. These codes represent the top and bottom 20% of the stroke codes according to their location estimates. The values of the discrimination parameters for the stroke codes fell within the range 0.5 to 2.5 suggesting the stroke codes generally present an acceptable level of discrimination [14]. The mean of the discrimination parameters was 1.020 (SD = 0.390). Code location parameters ranged from 4.468 to 11.582. The mean of the code location parameters was 9.617 (SD = 1.711). A test of goodness of fit gave small chi-square and high P values for 80% of the items, indicating no evidence of lack of fit for a large majority of codes, apart from those with low location parameter values. There was a strong negative relationship between code discrimination and location parameters, implying that highly discriminating codes tended to be less commonly recorded and vice versa. There was a weaker association between codes' location parameters and frequency.

**Table 3:** Estimated item parameters for a subsample (n = 28) of Read stroke codes retained for the IRT analyses.

Stroke code	Discrimination	SE	Location	SE	χ^2^
Thrombosis cavernous sinus	2.785	0.772	4.468	0.490	2136.3
Thrombosis transverse sinus	2.632	0.677	4.677	0.529	1934.8
Thrombosis of CNS venous sinuses NOS	2.077	0.635	5.717	1.007	816.9
CI due to cerebral venous thrombosis, nonpyogenic	1.642	0.446	5.998	1.162	653.7
Cortical haemorrhage	1.824	0.635	6.079	1.279	607.5
Phlebitis and thrombophlebitis of intracranial sinuses	1.813	0.648	6.265	1.458	518.2
Thrombosis lateral sinus	1.732	0.594	6.307	1.478	507.4
Vertebral artery occlusion	1.097	0.522	8.490	3.500	44.7
Subarachnoid haemorrhage from anterior	1.054	0.511	8.793	3.737	34.5
Brainstem infarction NOS	1.051	0.509	8.816	3.754	33.2
CI due to embolism of precerebral arteries	0.905	0.413	8.956	3.677	29.4
Pure motor lacunar syndrome	0.883	0.401	9.059	3.720	10.7
Ruptured berry aneurysm	0.797	0.341	9.177	3.595	7.7
Brainstem infarction	0.783	0.334	9.242	3.613	7.0
Thrombophlebitis of CNS venous sinuses	0.836	0.387	10.002	4.269	4.1
Extradural haemorrhage - nontraumatic	0.843	0.392	10.006	4.283	4.1
Subacute confusional state of cerebrovascular origin	0.811	0.371	10.007	4.227	3.9
Subarachnoid haemorrhage NOS	0.805	0.367	10.012	4.219	3.8
Left sided intracerebral haemorrhage, unspecified	0.850	0.396	10.013	4.299	4.1
Pontine haemorrhage	0.794	0.360	10.024	4.205	3.7
Intracerebral haemorrhage, intraventricular	0.774	0.347	10.059	4.185	3.4
CI due to unspecified occlus of precerbral arteries	0.880	0.417	10.079	4.387	4.0
Infarction of basal ganglia	0.760	0.339	10.091	4.174	3.2
Pure sensory lacunar syndrome	0.897	0.429	10.153	4.456	3.8
Subarachnoid haemorrhage from middle artery	0.735	0.323	10.163	4.161	2.8
Sequelae of stroke not specified	0.706	0.305	10.266	4.154	2.3
Occlusion and stenosis of middle cerebral artery	0.948	0.465	10.696	4.835	5.7
Occlusion and stenosis of posterior cerebral artery	0.972	0.483	11.582	5.384	0.0

## Discussion

This paper explored the feasibility and utility of a potentially novel application of item response theory. In the context of electronic patient records, item response theory models characterise the probability of a general practitioner recording, or not recording a stroke code that is drawn from a set of Read medical codes, as a function of the latent trait measured by these codes. In the present context, the latent trait may be regarded as reflecting the degree of confidence in a diagnosis of stroke which may range from low to high probability (ie the probability of endorsing a READ code given the underlying pathological stroke event as recorded by the GP). Usually the less frequently affirmed items give higher thresholds consistent with higher trait levels, so the frequently used codes would be associated with less certainty. Utilisation of stroke codes with higher parameters may then be viewed as enhancing the assertion that a genuine stroke event has occurred. Gulliford et al. has documented that READ medical codes could be reasonably placed on a continuum from low to high inter-rater agreement as to whether a genuine stroke event occurred [1]. Estimated discrimination and location parameters may have a potential utility in illustrating how different READ codes are used by GPs, or a requirement to improve diagnostic recording of clinical events in EPRs.

In this empirical study, the code location parameters of included stroke codes were generally high suggesting that each of these codes were associated with high trait levels [15]. Discrimination parameter estimates ranging from 0.67 to 2.79 consistent with the heterogeneity of stroke code content [16]. Restricted ranges of code location parameters and extreme location values have been previously reported in clinically-related IRT analyses [17-20]. The two-parameter IRT model gave more satisfactory fit than either the one- or three-parameter models but high chi-square values for about a fifth of the codes suggesting that the model fits less well for these codes. However, given the large size of the present sample, even marginal departure from the overall model may lead to an interpretation of model misfit [21]. It is advisable to be cautious in assessing the overall fit of the model.

In the present dataset, the most frequently used codes were indicative of non-specific stroke diagnoses (for example, 'cerebrovascular accident'). These codes could not be calibrated and, to the extent that these were excluded from estimation, the 2-PL model might be interpreted as identifying utilisation of these codes as aspects of stroke diagnosis recording where measurement is less precise and in need of improvement [7]. Poorly fitted stroke codes provide insights into the type of problems that system code developers should be wary of when introducing new stroke codes including duplicate and ambiguous codes. Adding new stroke codes into EPRs without avoiding duplication leads to unproductive workload for both practitioners and health service researchers. Detailed recording of stroke events in the EPRs depends on timely and accurate exchange of diagnostic information, including imaging results, between primary and secondary care providers, and this has to be prioritised if EPRs are to fulfil their potential.

Ideally, there should be limited variation in the use of Read codes between family practices. The present results indicate that this is not the case. One explanation may relate to the quality of information available to general practitioners when they select codes. For example, diagnostic information may be communicated from secondary care where there may be variation in the utilisation of imaging techniques to confirm stroke subtypes [15]. However, some general practitioners (GPs) may use free text to record additional details concerning stroke type after selecting a code that has limited clinical specificity. The implication of this finding is that the coding of stroke events in primary care might benefit from improved inter-agency communication (ie from secondary to primary care professionals).

### Limitations

Several considerations are necessary in interpreting the study findings. Firstly, the most frequently employed 33 stroke codes, accounting for a high proportion of participants, were excluded from calibration because the 2-PL model does not assign scale scores to participants with extreme response patterns on the stroke codes. If a code provides information of clinical relevance then it should not be excluded because it offers poor discrimination or has a particularly low location parameter [22]. However, if a code is very frequent but does not permit a detailed understanding of the stroke presentation then it should be revised [23]. The implication of this idea for this study is that the noncalibrated stroke codes should not be discarded without further input from clinical experts. The content or utilization of these codes should be revised consistent with the patient' stroke pathology in order to offer a more explicit differential diagnosis. Such endeavour would offer the opportunity to develop a pathological spectrum of stroke, for instance, from transient ischemic attacks (TIA) through degrees of varying pathology to 'pure' hemorrhagic or ischemic stroke.

Although the 2-PL model gave a better fit than the 1-PL or 3-PL models and showed satisfactory convergence with acceptable item fit statistics for the majority of codes, high standard errors were observed for estimated parameters for some of the stroke codes. The standard errors were particularly high among the codes at the extreme end of the continuum implying that the estimates for these codes may be less precise. This finding is consistent with previous suggestions that the use of large and rather homogeneous samples can result in highly precise estimates, but only for a limited range of the underlying latent trait [24]. Item information tends to vary by underlying trait level the estimates may be quite precise for some items and not so precise for others [7], as found in the present study. All standard errors, however, were an order of magnitude smaller than the parameter estimates.

The high chi-square values for some of the estimated code parameters also raises a question concerning the fit of the model for about a third of the stroke codes. However, fit statistics are susceptible to inflated Type I error rates due to grouping respondents into intervals based on their trait levels which contain error [25]. It was also asserted that the mechanical omission of misfiting items based on chi-square values or residuals alone, can improve the fit of the model as a whole, but worsens the fit of the remaining items [26]. Thus it is preferable to compare the fit of different models, rather than using chi-square to test the fit of one model [24]. In the present study, the 2-PL model fitted better the data than the 1-PL or 3-PL model implying that the present model represents a reasonable fit to the study data.

The assumptions of unidimensionality and local independence were not tested directly. The main reason for this was that applying factor analysis to categorical data can lead to distorted true factor structure and biased factor loadings [27]. Also several studies have shown that IRT models are rather robust to the violation of the unidimensionality assumption [28-30]. Further, in view of the fact that all stroke codes endorse a particular stroke event and that the endorsement of stroke codes is independent of each other it is highly probable that the assumptions of unidimensionality and local independence are upheld by the data. These would be interesting to be confirmed in future studies, however.

Notwithstanding above limitations, following Reise [31] the present analysis is illustrative in highlighting new challenges for standard IRT models when applied to clinical populations. Secondly, negative research studies are rarely published despite the fact that these have the potential to stimulate further developments in the field.

Future work along the lines of the present study would lead to a better understanding of which IRT models are better positioned to validate the diagnosis of stroke events in EPRs. For instance, in the present study the 2-PL provided a better fit to the data compared to the 1-PL or 3-PL models. However, further research should extend the analyses to multidimensional or unidimensional models with four parameters.

## Conclusions

This study exemplifies the potential application of IRT analysis to understanding the utilisation of different medical codes by GPs to discriminate between different stroke events and possibly to optimize future registering of stroke events within EPRs. Several methodological barriers and limitations have been identified that require addressing through future research.

## Competing interests

The authors declare that they have no competing interests.

## Authors' contributions

MG, AG, and TS obtained the data. MG and AD conceived and designed the study. AD performed the statistical analysis. All authors helped draft the manuscript, read and approved the final manuscript.

## Pre-publication history

The pre-publication history for this paper can be accessed here:

http://www.biomedcentral.com/1471-2288/11/168/prepub
